# Influence of Environmental Factors on the Starch Quality of Sorghum: A Multifaceted Analysis of Structural, Nutritional, and Functional Profiles

**DOI:** 10.3390/foods14244204

**Published:** 2025-12-07

**Authors:** Fulai Ke, Baizhi Chen, Kuangye Zhang, Jiaxu Wang, Linlin Yang, Zeyang Zhao, Fei Zhang, Han Wu, Zhipeng Zhang, Feng Lu, Yanqiu Wang, Youhou Duan, Zhiqiang Liu, Jianqiu Zou, Kai Zhu

**Affiliations:** Sorghum Research Institute, Liaoning Academy of Agricultural Sciences, Shenyang 110161, China; fulaike1981@163.com (F.K.); chenbaizhi0307@163.com (B.C.); zky1319577703@163.com (K.Z.); w15640044750@163.com (J.W.); yanglinlin0330@163.com (L.Y.); zhaozeyangzz@163.com (Z.Z.); zhangfei19821121@163.com (F.Z.); wuhan8453@sina.com (H.W.); zzp906@163.com (Z.Z.); lufeng720202023@163.com (F.L.); wangyanqiu73@126.com (Y.W.); duanyouhou@163.com (Y.D.); baizhichen1996@gmail.com (Z.L.); jianqiuzou@126.com (J.Z.)

**Keywords:** sorghum, starch structure, environmental factors, grain quality

## Abstract

Understanding how environmental factors modulate starch structure and functionality in sorghum is critical for optimizing its application in the food processing and fermentation industries. In this study, two sorghum cultivars with distinct starch types—Liaonian 3 (LN3, waxy) and Liaoza 82 (LZ82, non-waxy)—were cultivated across four major ecological regions in China to systematically investigate the combined effects of temperature and precipitation on grain composition, starch molecular structure, and processing properties. Comprehensive analyses, including scanning electron microscopy, molecular weight profiling, chain-length distribution, crystallinity, molecular order, and thermal/pasting behaviors, demonstrated that precipitation is the predominant environmental factor driving starch biosynthesis and structural assembly. High precipitation levels promoted amylopectin accumulation, shorter chain formation, increased branching degree, and higher crystallinity and molecular order, ultimately enhancing starch thermal stability and paste consistency. Genotypic differences further modulated starch structural patterns and environmental responsiveness, with LN3 consistently exhibiting higher amylopectin content, crystallinity, double-helix proportion, and gelatinization enthalpy compared to LZ82. Correlation analyses revealed genotype-dependent regulatory relationships linking environmental cues to starch structure and processing functionality. These findings provide a comprehensive framework elucidating the environmental regulation of starch structure–function relationships in sorghum, offering theoretical insights for climate-resilient breeding and functional starch development.

## 1. Introduction

Sorghum (*Sorghum bicolor* (L.) Moench) is a grass species belonging to the Poaceae family and is ranked as the sixth most widely cultivated crop globally, following maize, wheat, rice, soybean, and barley. In 2023, the global harvested area reached 39,851,368 hectares (www.fao.org). As a drought-tolerant and salt- and alkali-resistant crop, sorghum serves various purposes, including food, animal feed, alcoholic beverages, and industrial processing [1,2]. In China, sorghum is primarily used as a raw material for liquor production, particularly for baijiu (Chinese distilled liquor) [3]. The nutritional components of sorghum grains, such as starch, fat, protein, and tannins, significantly influence both the yield and quality of baijiu. Starch, which constitutes about 55–75% of the sorghum grain, is the main component and has a profound effects on the alcohol yield as well as flavor profile of the liquor [4,5]. The total starch content, along with the ratio of amylopectin to amylose and resistant starch, plays a key role in determining both the fermentation yield and the sensory qualities of the final product [6].

In an increasing number of studies on plant starch, researchers have found that its physical structures, such as crystallinity, double-helix vs. single-helix structure, degree of order, amorphous regions, granule size, and morphology, significantly influence its processing properties [7]. For example, in noodle processing, dry and cooked noodles made from small-sized starch granules (<20 μm) exhibited superior processing performance and quality, as assessed by both objective and subjective methods, compared to those made from larger granules [8]. Additionally, starch crystallinity has been shown to primarily contribute to bread firmness [9]. Encouragingly, research on the relationship between starch structure and baijiu production in sorghum has also begun to emerge. The crystalline structure of starch influences baijiu flavor and brewing efficiency by altering the amount and structure of leached starch [10]. Similarly, the high crystallinity and gelatinization enthalpy in sorghum grains reduce cooking resistance by enhancing swelling power, which facilitates breaking the seed coat and cell wall, thereby affecting both alcohol yield and baijiu quality [11]. Thus, research on starch structure has become a growing focus in efforts to improve both the alcohol yield and the quality of baijiu.

The starch content and structure of crop grains are not solely determined by genetic factors but are also significantly influenced by the interaction between genetics and environmental conditions. For example, high-temperature stress has been shown to increase the average granule size of starch in waxy maize and enhance the relative crystallinity percentage in rice starch [12,13,14]. Similarly, rice grown under controlled low-temperature environments exhibited an increased ratio of amylose to amylopectin [15]. Moderate water deficits in cereals can reduce starch accumulation by up to 40%, altering starch composition, structure, and functionality [16]. Drought stress significantly decreases the amylose content in wheat and rice grains [17,18], while also modifying the starch granule size distribution in wheat. Specifically, drought conditions lead to a higher proportion of A-type granules, accompanied by reductions in B- and C-type granules [19].

Global climate change exacerbates the challenges faced by agriculture, with rising temperatures, frequent droughts, and flooding events having profound impacts on global food production and quality [20]. Despite these challenges, research on sorghum grain quality—particularly starch content and structure—has predominantly been conducted within single geographic regions [21,22,23,24]. This regional limitation hampers a comprehensive understanding of how environmental changes influence sorghum starch properties, making it difficult to develop strategies to address the complexities of future climate scenarios. To address this gap, the present study selected two representative sorghum cultivars—Liaonian 3 (LN3, waxy type) and Liaoza 82 (LZ82, non-waxy type)—and conducted field trials across four major sorghum-producing regions in China. This multi-environmental study aimed to systematically evaluate the effects of environmental variation on sorghum grain quality, with a particular focus on starch content and structural characteristics. Building on this framework, we hypothesize that climatic factors such as temperature and precipitation regulate starch molecular organization and grain nutrient composition, thereby affecting functional properties such as gelatinization and pasting behavior.

## 2. Materials and Methods

### 2.1. Plant Materials and Experimental Design

Two sorghum (*Sorghum bicolor* L. Moench) cultivars—Liaonian 3 (LN3, waxy) and Liaoza 82 (LZ82, non-waxy)—were provided by the Sorghum Research Institute of the Liaoning Academy of Agricultural Sciences. Field trials were conducted in four representative sorghum-producing regions in China, each corresponding to a distinct ecological zone: (1) an early-maturing spring sowing region in Chifeng (42.27° N, 118.90° E), Inner Mongolia; (2) a late-maturing spring sowing region in Lvliang (37.52° N, 111.13° E), Shanxi Province; (3) a spring-summer dual-sowing zone (spring and summer) in Jinan (36.65° N, 117.12° E), Shandong Province; and (4) a southern planting region in Guiyang (26.65° N, 106.63° E), Guizhou Province. Mature grains were harvested, conditioned to a standard moisture content of 14%, and then milled using a laboratory hammer mill to ensure consistent particle size for subsequent analyses.

All field experiments were conducted using a randomized complete block design (RCBD) with three replicates. Identical field management practices were applied across all locations. Basal fertilization was performed once prior to sowing, including nitrogen (200 kg/ha), phosphorus (80 kg/ha), and potassium (50 kg/ha).

### 2.2. Determination of Grain Nutritional Components

Starch content was determined directly from sorghum flour samples using enzymatic hydrolysis followed by the glucose oxidase–peroxidase (GOPOD) assay [25]. Amylose content (AC) was measured using a dual-wavelength colorimetric method [3]. Protein content was quantified by the Kjeldahl method [26]. The resistant starch (RS) content was determined using the enzymatic digestion method described in AOAC Official Method 2002.02. Tannin content was analyzed according to the procedure described by Ebadi et al. [27]. Lipid content was determined using Soxhlet extraction following AOAC Official Method 920.39—Fat (Crude) in Cereal Products. The content of amylopectin was calculated as the total starch content minus the amylose content [28].

### 2.3. Starch Sample Preparation

Starch from sorghum seeds (two cultivars) was isolated by alkaline steeping. Dehulled sorghum kernels were milled and immersed in 3 g L^−1^ NaOH at 35 °C for 12 h. The slurry was passed sequentially through 100- and 200-mesh screens and centrifuged at 3000× *g* for 10 min. After discarding the supernatant, the yellowish residues were removed to obtain a white starch pellet, which was resuspended in distilled water, recentrifuged, neutralized with 0.1 mol L^−1^ HCl, dried at 40 °C, and finally sieved through 100-mesh.

### 2.4. Scanning Electron Microscopy

Starch samples were processed as follows: the extracted starch was ground, dispersed, and passed through a 100-mesh sieve. Approximately 0.1 g of the sieved starch was weighed and transferred into a 2 mL screw-cap tube. To the tube, 1 mL of 4% SDS-wash buffer was added and vortexed thoroughly. The mixture was centrifuged at 10,000× *g* rpm for 1 min, and the supernatant was discarded. This washing step was repeated twice. Next, 1 mL of deionized water was added, vortexed, and centrifuged at 10,000× *g* rpm for 1 min, with the supernatant discarded; this step was repeated five times. Finally, 1 mL of absolute ethanol was added, vortexed, and mixed thoroughly.

A small amount of the prepared starch suspension (~200 μL) was pipetted onto a conductive adhesive on a copper stage, evenly spread, and left to dry overnight at 37 °C. The dried sample was coated with gold using an ion sputter coater and examined using a Zeiss Merlin Compact scanning electron microscope (Carl Zeiss, Oberkochen, Germany). Images were obtained under an accelerating voltage (EHT = 3.00 kV), working distance (WD = 8.5 mm), and probe current (I = 0.1 pA), using a secondary electron detector (Signal A = SE2) at a magnification of 2000×.

### 2.5. Determination of Granule Size

One hundred milligrams of starch were ultrasonically blended in 1 mL of 75% ethanol. Granule size was estimated using a Mastersizer 3000 laser diffraction particle size analyzer (Malvern Instruments Ltd., Worcestershire, UK) at a range of 0.1–3500 μm. The number-, volume-, and surface-weighted mean diameters, and the 10th percentile (d(0.1)), median (d(0.5)), and 90th percentile (d(0.9)) of volume were calculated following the protocol previously described by Lin et al. [29].

### 2.6. Determination of Starch Molecular Weight

Starch samples (5 mg) were dissolved in 5 mL of 0.5% LiBr/DMSO at 80 °C for 3 h. Molecular weight distribution was analyzed using a size exclusion chromatography system equipped with multi-angle laser light scattering (MALLS) and refractive index detectors (SEC-MALLS-RI). Separation was performed on a series of Ohpak columns at 60 °C using isocratic elution with DMSO (0.5% LiBr) at 0.3 mL/min. Data were processed with ASTRA 6.1 software to calculate weight-average molecular weight (Mw), number-average molecular weight (Mn), Z-average molecular weight (Mz), Root-mean-square radius of gyration (Rz) and polydispersity index (PDI).

### 2.7. Chain-Length and Branching Analysis of Starch

The chain-length distribution of debranched amylopectin was analyzed using high-performance anion-exchange chromatography (HPAEC) with pulsed amperometric detection (PAD), performed on a Dionex ICS5000+ system (Thermo Fisher Scientific, USA) equipped with a CarboPac PA-200 column (250 × 4.0 mm, 10 μm). The mobile phase consisted of 0.2 M NaOH (solvent A) and 0.2 M NaOH with 0.2 M sodium acetate (solvent B). The flow rate was 0.4 mL/min, with an injection volume of 5 μL. The gradient program was as follows: 0–10 min, A:B = 90:10 (*v*/*v*); 10–30 min, linear gradient to 40:60 (*v*/*v*); 30–50 min, maintained at 40:60 (*v*/*v*); 50.1–60 min, re-equilibrated to 90:10 (*v*/*v*).

The branching characteristics of starch were analyzed using proton nuclear magnetic resonance (^1^H NMR). Approximately 5 mg of purified starch was dissolved in 1 mL of D_6_-DMSO by heating at 80 °C overnight. After centrifugation at 12,000× *g* rpm for 10 min, the supernatant was transferred to an NMR tube for measurement. Spectra were acquired on a Bruker BioSpin ^1^H NMR spectrometer (500.23 MHz) with 32 scans. Data were processed using MestReNova software 15.1.0 to evaluate the degree of branching based on characteristic chemical shifts.

### 2.8. X-Ray Diffraction and Fourier-Transform Infrared (FTIR) Analysis

The crystal type and relative crystallinity of starch granules were analyzed using an X-ray diffractometer (XRD; D/Max2550VB+/PC, Rigaku Corporation, Tokyo, Japan) following the method described by Wang et al. [30]. The samples were scanned using Cu Kα radiation (λ = 0.15406 nm) at 40 kV and 40 mA over a 2θ range of 4–60°, with a step size of 0.02° and a scanning speed of 4°/min. Crystallinity and peak patterns were analyzed using MDI Jade 5.0 software.

Fourier-transform infrared spectroscopy (FTIR; Nicolet iZ-10, Thermo Fisher Scientific, Waltham, MA, USA) was used to evaluate the short-range molecular order of starch. Dried starch samples were mixed with KBr and pressed into pellets. Spectra were collected in the range of 4000–400 cm^−1^ with a resolution of 4 cm^−1^ and 32 scans per sample.

### 2.9. Structural Characterization of Starch by Solid-State ^13^C NMR

The molecular order of starch was analyzed using solid-state ^13^C CP/MAS NMR spectroscopy (Bruker AVANCE III 400 WB, Bruker BioSpin GmbH, Rheinstetten, Germany). Starch samples were gelatinized, freeze-dried, ground, and moisture-equilibrated in saturated NaCl before packing into a 7 mm rotor. Spectra were acquired at a ^13^C resonance frequency of 100.6 MHz with a MAS rate of 6 kHz, contact time of 1.2 ms, and a relaxation delay of 2.0 s. A total of 1000–1600 scans were accumulated. Spectral deconvolution of the C1 region was performed using PeakFit software version 4.2 to quantify amorphous, single-helix, and double-helix structures.

### 2.10. Thermal and Pasting Property Analysis of Starch

Thermal properties of starch samples were measured using a differential scanning calorimeter (DSC) (Q2000, TA Instruments, New Castle, DE, USA; or DSC 200 F3, NETZSCH, Selb, Germany). Approximately 3 mg of starch and 6 μL of distilled water were sealed in an aluminum pan and stored at 4 °C overnight. An empty sealed pan was used as the reference. The sample was heated from 30 °C to 105 °C at a rate of 10 °C/min. Thermal transitions were analyzed using Universal Analysis version 5.5 or Proteus Thermal Analysis software version 9.0, peak temperature (Tp) and gelatinization enthalpy (ΔH) were obtained from the thermograms to characterize the phase transition behavior.

Approximately 100 mg (±1 mg) of starch sample (adjusted to 12% moisture basis) was weighed into a 15 mL glass tube. After adding 0.2 mL of 0.025% thymol blue indicator and 2 mL of 0.2 mol/L KOH, the mixture was vortexed thoroughly. The sample was gelatinized in a boiling water bath for 8 min with a glass bead covering the tube. After gelatinization, the sample was cooled at room temperature for 5 min and further chilled in an ice bath for 20 min. The tube was then placed horizontally at 25 ± 2 °C for 1 h. The flow length (mm) of the starch paste was recorded to evaluate its paste consistency.

### 2.11. Statistical Analysis

All experiments were performed in triplicate. Statistical analyses were conducted by one-way analysis of variance (ANOVA) followed by Duncan’s multiple range test using SPSS 23.0. Differences at *p* < 0.05 were considered statistically significant. Graphical outputs were generated with Origin 2022. PCA and RDA were performed using the prcomp function and the vegan package in R version 4.5.0, and correlation analysis was conducted using the corrplot package [31,32].

## 3. Results and Discussion

### 3.1. Effects of Environmental Factors on the Composition of Sorghum Seeds

**Figure 1:**
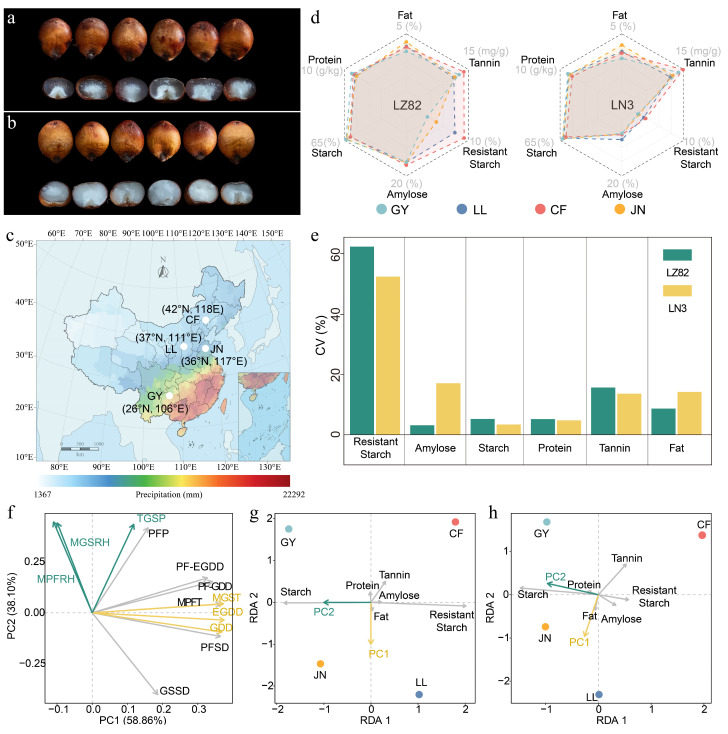
Effects of environmental factors on the composition of sorghum seeds. (**a**,**b**) Whole-seed and transverse-section images of LZ82 and LN3 grains. (**c**) Geographic locations of experimental sites in four major sorghum ecological zones. On the map, colors from blue to red represent precipitation levels from low to high. (**d**) Contents of various grain quality traits in LZ82 and LN3. (**e**) Coefficients of variation (CV) for grain-quality traits in LZ82 and LN3. (**f**) PCA of environmental variables across experimental sites, including mean growing-season air temperature (MGST), mean growing-season relative humidity (MGSRH), total growing-season precipitation (TGSP), growing-season sunshine duration (GSSD), growing degree days (GDD), effective growing degree days (EGDD), mean pre-flowering air temperature (MPFT), mean pre-flowering relative humidity (MPFRH), total post-flowering precipitation (PFP), post-flowering sunshine duration (PFSD), post-flowering cumulative growing degree days (PF-GDD), and post-flowering effective growing degree days (PF-EGDD). (**g**) RDA between principal components of environmental factors and seed composition-related traits in LZ82. (**h**) RDA between principal components and seed composition-related in LN3. The arrows represent environmental/starch variables, and their directions and lengths indicate the strength and direction of correlations.

To ensure the representativeness and generalizability of the experimental materials, this study selected two sorghum genotypes—the non-waxy sorghum LZ82 and the waxy sorghum LN3—which share similar growth durations. Field trials were conducted in four representative locations across major sorghum agroecological zones in China: the early-maturing spring-sowing zone (Chifeng, CF), the late-maturing spring-sowing zone (Lvliang, LL), the spring-summer dual-sowing zone (Jinan, JN), and the southern zone (Guiyang, GY) (Figure 1a,c). Grain quality assessments demonstrated strong environmental influences on starch-related traits (Figure 1d; Table 1). The coefficients of variation (CV) for resistant starch were 62.19% in LZ82 and 52.33% in LN3, with resistant starch contents ranging from 1.76% to 9.17% in LZ82 and 0.73% to 2.32% in LN3 (Figure 1e), highlighting the existence of strong genotype-environment interactions. Across all regions, amylose content ranged from 15.1% to 16.2% in the non-waxy cultivar LZ82 and from 5.1% to 7.2% in the waxy cultivar LN3, confirming the clear genotypic distinction in starch composition. Notably, sorghum grains cultivated in Guiyang tended to have the highest total starch contents but lower levels of amylose and resistant starch. This high total starch–low resistant starch profile enhances digestibility for both humans and livestock and improves fermentation efficiency, which underscores sorghum’s role as a premium raw material for liquor production in Guizhou, particularly for Maotai liquor. Thus, the unique agroecological conditions of Guizhou are conducive to high-quality sorghum production, providing significant economic value [33]. To better characterize these agroecological differences, key climatic variables were recorded throughout the growing season at each site (Table 2). The mean growing-season temperature (MGST) ranged from 21.7 °C in GY to 26.4 °C in JN, while total growing-season precipitation (TGSP) varied widely from 184 mm in CF to 585 mm in JN. Relative humidity during grain filling was highest in GY (77.3%) and lowest in CF (47.7%). Growing degree days (GDD) and effective GDD (EGDD) also showed substantial spatial variation, with JN exhibiting the highest accumulated values (3110 °C and 1930 °C, respectively). These results indicate that the four experimental sites provided distinct thermal and hydrological conditions, representing the main climatic gradients of sorghum-producing regions in China.

To further assess how these climatic differences influenced grain quality, all environmental variables listed in Table 2 were standardized and incorporated into principal component analysis (PCA) and redundancy analysis (RDA). The PCA revealed that the first two components explained 97.96% of the phenotypic variation (PC1 = 58.86%; PC2 = 38.10%). PC1 was predominantly influenced by temperature-related factors such as MGST, EGDD and GDD, whereas PC2 was primarily associated with precipitation-related variables, including mean pre-flowering relative humidity (MPFRH), mean growing-season relative humidity (MGSRH) and TGSP (Figure 1f). RDA showed that, for LZ82, the first two RDA axes explained 94.53% of the trait variation (RDA1 = 90.28%, RDA2 = 4.25%). PC2 exhibited a significant positive correlation with total starch content, while showing significant negative correlations with both amylose and resistant starch content (Figure S1a). In LN3, the first two RDA axes jointly explained 70.16% of trait variation (RDA1 = 56.25%, RDA2 = 13.91%), where PC2 was also significantly positively correlated with total starch content and negatively correlated with resistant starch content (Figure S1b). Combined with the PCA results, where PC2 was largely associated with precipitation-related variables, these findings indicate that precipitation may be an important environmental factor influencing sorghum grain quality, particularly starch composition. This may be attributed to the fact that adequate water availability significantly enhances the activities of key starch biosynthesis enzymes, including ADP-glucose pyrophosphorylase (ADPG-PPase), UDP-glucose pyrophosphorylase (UDPG-PPase), soluble starch synthase (SSS), and granule-bound starch synthase (GBSS). Conversely, post-anthesis drought stress may elevate abscisic acid (ABA) levels and reduce indole-3-acetic acid (IAA) concentrations, thereby inhibiting the activities of starch synthases and ultimately affecting grain development [34]. In addition to starch traits, other compositional parameters also showed clear genotype–environment interactions (Table 1). Protein content exhibited an opposite trend, increasing slightly under humid conditions (e.g., 8.54 g kg^−1^ in GY) compared with drier sites. Fat (2.84–4.29%) and tannin (10.65–14.81 mg g^−1^) contents fluctuated modestly among sites but tended to be higher under thermal or mild stress conditions. These results indicate that climatic factors such as temperature and precipitation jointly shape both starch structure and nutritional composition [35].

### 3.2. Starch Granule Morphology and Size of Different Varieties Across Regions

**Figure 2:**
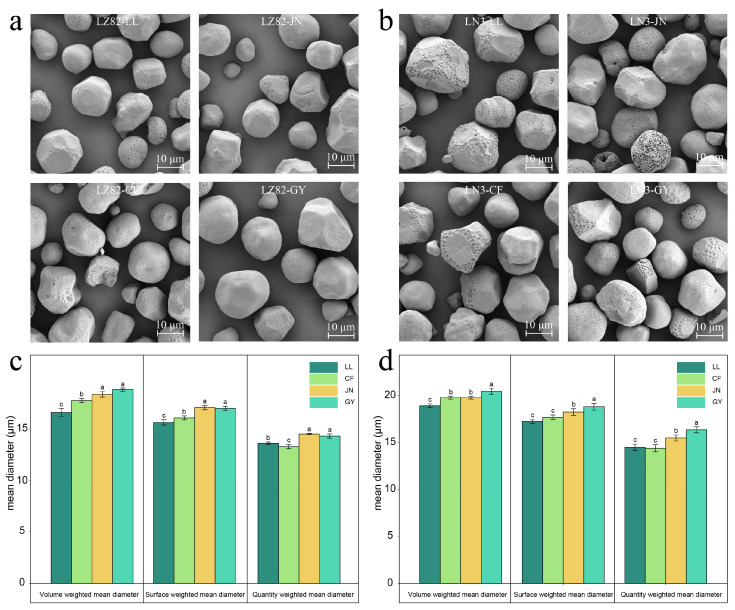
Starch granule morphology and size distribution. (**a**,**b**) SEM images of starch from two sorghum varieties grown in four different ecological regions. (**c**) Particle size distribution of LZ82 starch under different environmental conditions. (**d**) Particle size distribution of LN3 starch under different environmental conditions. Different lower-case letters indicate statistical significance at *p* < 0.05.

Environmental factors, including precipitation, temperature, and fertilization practices, significantly regulate the morphological characteristics of starch granules [36]. Systematic observations using scanning electron microscopy (SEM) revealed that, although sorghum grains cultivated in different ecological regions exhibit consistent starch granule morphologies—primarily irregular spherical or polygonal structures (Figure 2a,b)—distinct phenotypic traits are evident among different genotypes. Specifically, the LZ82 variety displays more regular spherical shapes with smooth surface structures, whereas the LN3 variety exhibits features resembling golf ball-like surface depressions or pinhole-like pores. Studies have confirmed that these surface indentations are connected to internal cavities through tubular channels, which may increase the enzyme-substrate contact area, thereby enhancing the starch digestion efficiency in the LN3 variety [37,38]. This mechanism also provides an explanatory basis for the biological characteristics of waxy sorghum as a high-quality feedstock [39].

Starch granule size distribution is a key physicochemical parameter that determines its functional properties and suitability for industrial applications [40]. In this study, sorghum starch exhibited a typical granule size range of 11–30 μm (Table S1), which is broader than that of corn starch (5–25 μm) [41]. Regional climate analysis revealed that samples from GY and JN had significantly higher values for d(0.1) and d(0.5), as well as for the number-, volume-, and surface area-weighted mean diameters, compared to those from LL and CF (Figure 2b,c; Table S1). These findings suggest that relatively humid environmental conditions during the growing season promote the full development of starch granules [34]. At the cultivar-specific level, under the same cultivation conditions, the volume-weighted mean diameter (VWMD) of starch granules in waxy sorghum (LN3) ranged from 18.90 to 20.43 μm, which was consistently larger than that of the non-waxy sorghum (LZ82), ranging from 16.63 to 18.87 μm (Table S2).

### 3.3. Starch Molecular Weight Profiles of Different Varieties Across Regions

As shown in Table 3 and Figure S2, sorghum samples exhibited clear regional variability in several molecular parameters, including number-average molecular weight (Mn), weight-average molecular weight (Mw), z-average molecular weight (Mz), polydispersity index (PDI), and root-mean-square radius (Rz). In absolute molecular weight analysis, the waxy sorghum variety LN3 consistently showed higher Mw than the non-waxy variety LZ82 across all ecological zones (Table 3), which is attributed to its higher amylopectin content and more complex branched molecular architecture [26,42]. However, in the GY region, the difference in weight-average molecular weight (Mw) between LN3 and LZ82 was notably reduced, with values of 13.30 × 10^7^ g/mol for LN3 and 12.88 × 10^7^ g/mol for LZ82. Their number-average molecular weights (Mn) were nearly identical, 7.25 × 10^7^ g/mol for LN3 and 7.95 × 10^7^ g/mol for LZ82, with LZ82 slightly surpassing LN3—opposite to trends observed in other regions. Additionally, LZ82 from GY exhibited the lowest polydispersity index (PDI = 1.62) among all samples, indicating a narrower and more uniform molecular weight distribution, and the smallest Rz (283.47 nm), suggesting a more compact chain configuration [43]. Analysis of the z-average molecular weight (Mz) further highlighted regional differences: in GY, LN3 showed a slightly higher Mz (19.43 × 10^7^ g/mol) than LZ82 (17.66 × 10^7^ g/mol), but both varieties had noticeably lower Mz values compared to their counterparts in other regions. These results indicate that the higher precipitation in GY is associated with a shift in starch molecular-weight distribution, favoring fewer ultra-high-molecular-weight molecules and more intermediate-molecular-weight species.

Interestingly, as previously noted, LZ82 samples from GY and JN exhibited the highest total starch content. Their amylose and resistant starch levels were significantly lower than those of samples grown in CF, the site with the lowest precipitation (Table 1). This profile suggests that enhanced starch biosynthetic activity under high precipitation conditions may promote the assembly of abundant and uniform amylopectin chains, while reducing the accumulation of long, linear amylose molecules. Such structural features are consistent with the observed decreases in Mn, Mz, and PDI, reinforcing the notion that ecological factors—especially water availability—play a critical role in shaping starch molecular architecture (Table 3; Figure S2).

### 3.4. Fine Structure of Starch from Different Varieties Across Regions

**Figure 3:**
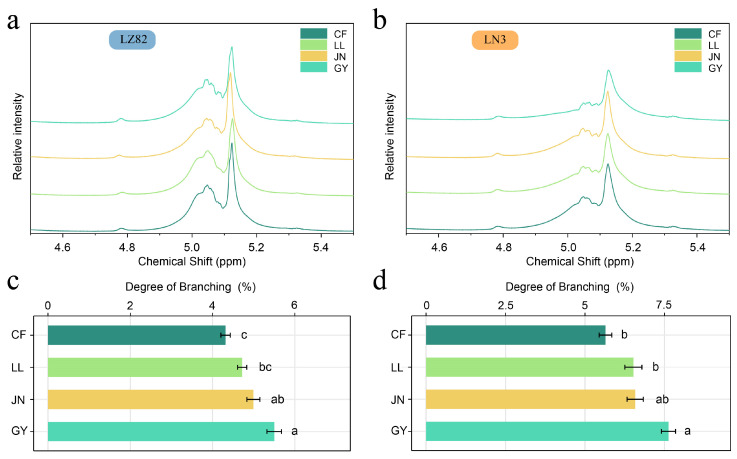
Effects of different ecological environments on the degree of branching of sorghum grain starch. (**a**,**b**) Representative ^1^H NMR spectra of LZ82 and LN3; (**c**,**d**) Degree of branching in LZ82 and LN3 starch from different ecological regions. Different lowercase letters indicate statistically significant differences at *p* < 0.05.

The chain-length distribution and degree of branching (DB) are critical parameters reflecting the fine architecture of amylopectin, which profoundly influence starch molecular conformation, crystallization behavior, thermal stability, and processing characteristics [44]. Amylopectin chains are commonly classified into four types based on their degree of polymerization (DP): A chains (DP 6–12), B1 chains (DP 13–24), B2 chains (DP 25–36), and B3 chains (DP ≥ 37) [45]. In this study, the waxy sorghum variety LN3 exhibited significantly higher proportions of A and B1 chains compared to the non-waxy variety LZ82, whereas LZ82 showed an enrichment of longer B2 and B3 chains (Figure 3a,b, Table S2). This structural divergence is largely attributed to genetic background: LN3 is almost entirely composed of amylopectin, allowing starch branching enzymes (SBEs) to function without amylose interference, resulting in more frequent and ordered short-chain formation [46]. In addition, debranching enzymes (DBEs) cooperate with SBEs to remove improper branch linkages and refine the amylopectin architecture, thereby maintaining an optimal chain-length distribution [46]. In contrast, the higher amylose content in LZ82 may hinder the synthesis and alignment of medium and short branches, leading to an increase in long-chain segments.

**Figure 4:**
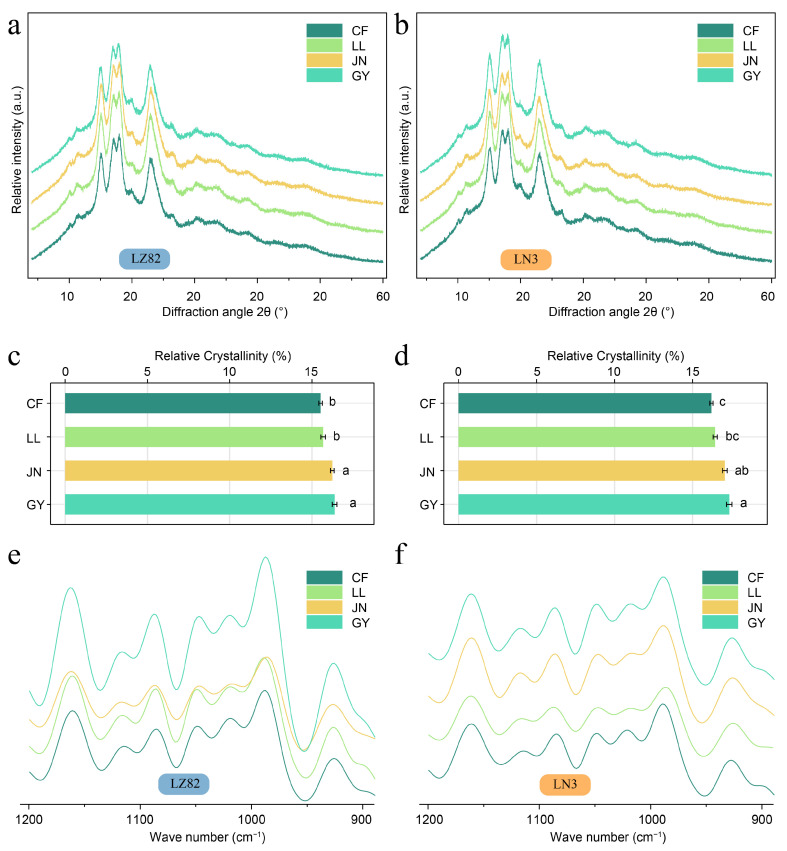
Effects of different ecological environments on the crystallinity and molecular order of sorghum grain starch. (**a**,**b**) XRD patterns of LZ82 and LN3 starch under different ecological conditions. (**c**) Proportion of relative crystallinity in LZ82 from different ecological regions. (**d**) Proportion of relative crystallinity in LN3 from different ecological regions. (**e**,**f**) FTIR spectra of LZ82 and LN3 starch under different ecological conditions. Different lower-case letters indicate statistical significance at *p* < 0.05.

Further analysis of the DB revealed that LN3 consistently exhibited a higher degree of branching than LZ82 across all ecological regions (Table S3). This pattern is closely associated with the higher proportions of A and B1 chains in LN3. A high degree of branching reflects a greater frequency of inter-chain linkages among glucan branches, which facilitates the formation of a dense chain network. Such a network provides a structural basis for the assembly of double helices and the development of crystalline regions [47]. This trend is consistent with subsequent observations that LN3 samples possess higher crystallinity and a greater proportion of double helices (Figure 4), suggesting that variations in chain length and branching patterns can directly drive the enhancement of starch macrostructural order [48].

In addition to genetic factors, environmental conditions play a significant role in modulating chain-length distribution and branching degree. In particular, in GY and JN—regions characterized by abundant precipitation—both LN3 and LZ82 exhibited higher proportions of A and B1 chains, along with an increased degree of branching (Figure 3c,d). The amylopectin content was also significantly elevated (Table S4). This may be attributed to enhanced enzyme activities involved in carbohydrate transport and synthesis under sufficient water availability, which promotes the synthesis of short-branched chains and contributes to increased branching complexity [34]. The combination of high branching degree and elevated proportions of short to intermediate chains supports the formation of a more stable molecular network, improving starch structural compactness and thermal stability [47,49]. In summary, sorghum starches exhibited substantial differences in chain-length structure and degree of branching among varieties and ecological environments. These fine structural features determine the foundation of crystalline architecture and influence the starch’s processing adaptability and functional performance. The coordinated regulation of chain length and branching provides a theoretical basis for understanding starch structure–function relationships and offers important insights for the development of functional starches and precision breeding of sorghum varieties.

### 3.5. Crystal Structure and Short-Range Molecular Order of Starch

Sorghum starch granules exhibit a semicrystalline structure, as evidenced by XRD patterns from samples grown in different ecological regions. Characteristic diffraction peaks appear at 2θ values of 15.1°, 17.2°, 17.9°, and 23.2°, with no peak observed at 5.6° (Figure 4a,b), indicating a typical A-type crystalline structure. This pattern remains consistent across diverse climatic conditions associated with different cultivation areas. Although some regional comparisons did not show statistically significant differences, starch from the more humid sites (e.g., JN and GY) still exhibited numerically higher mean relative crystallinity than that from the arid regions (CF and LL), and showed significant differences compared with CF, the site with the lowest precipitation. These patterns suggest that greater precipitation may promote starch crystallization (Table 2, Figure 4c,d). Relative crystallinity is primarily determined by amylopectin characteristics, including its content, branch chain length, crystal size, and double-helix arrangement, as well as the molecular interactions involved [50]. The crystalline regions are composed of double helices formed by the short B1 chains of amylopectin. The higher crystallinity observed in GY samples is consistent with their greater amylopectin and B1 chain content. Moreover, the waxy sorghum variety LN3 consistently exhibits higher relative crystallinity than the non-waxy variety LZ82, even when grown under the same conditions. LN3, with its higher amylopectin content, shows enhanced crystallinity compared to LZ82, as supported by the data in Table S5.

FTIR spectroscopy is effective in detecting short-range molecular order in starch [51]. As illustrated in Figure 4e,f, deconvoluted spectra of sorghum starches exhibit characteristic absorption bands from C–O and C–C stretching vibrations in the 1200–900 cm^−1^ region [52,53]. The absorbance ratio at 1047/1022 cm^−1^—commonly used to assess short-range order (DO)—reflects the degree of crystallinity near the granule surface, with values listed in Table S6. Notably, the starches from LN3 and LZ82 grown in GY show significantly higher 1045/1022 cm^−1^ ratios (0.99 and 1.01, respectively) than those from other regions, consistent with their elevated relative crystallinity. Short-range molecular ordering in starch arises from double-helix structures, which are affected by the conformation of starch chains. It has been reported that aggregated amylose can hinder the formation of double helices among amylopectin branches [26]. Consistently, the abundant amylopectin in LN3 promotes greater structural order.

### 3.6. Solid-State NMR Analysis of Starch Structure and Environmental Effects

**Figure 5:**
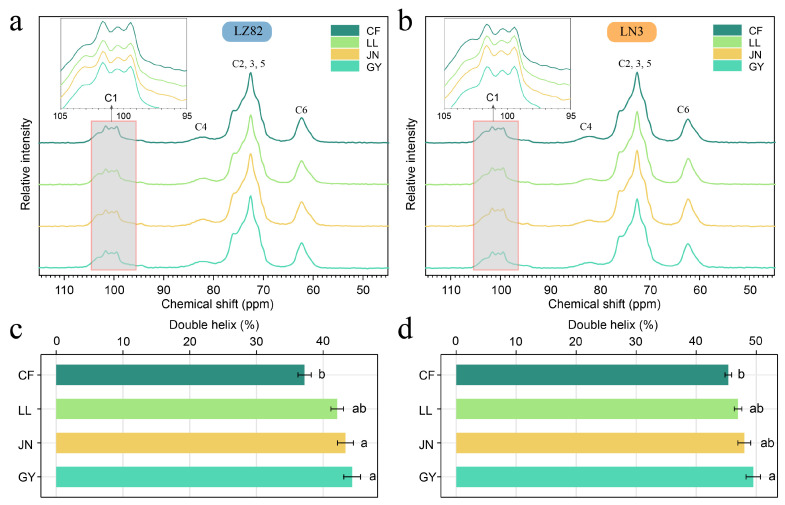
Helical structure analysis of starch samples from different regions. (**a**) ^13^C CP/MAS NMR spectra of LZ82 starches. (**b**) ^13^C CP/MAS NMR spectra of LN3 starches. (**c**) Proportion of double helices in LZ82 from different ecological regions. (**d**) Proportion of double helices in LN3 from different ecological regions. Different lower-case letters indicate statistical significance at *p* < 0.05.

The solid-state ^13^C CP/MAS NMR spectra of starches are presented in Figure 5, with resonance assignments based on prior studies [54]. This technique provides insights into the molecular organization of starch at short distance scale [55]. The C1 resonance reflects both crystalline and non-crystalline (but rigid) chain structures, and its multiplicity indicates the packing type of starch granules. Similarly to normal maize starch, sorghum starch exhibits a characteristic triplet at 99.5, 100.5, and 101.5 ppm (Figure 5a,b), confirming A-type crystallinity, which is consistent with X-ray diffraction results [29]. Although some regional comparisons were not statistically significant, samples from the regions with higher relative humidity (GY) still showed numerically higher proportions of double helices in both LN3 (49.50%) and LZ82 (44.36%) than those from the drier sites, suggesting a potential positive influence of relative humidity on double-helix formation (Figure 5c,d, Table S7). This suggests that increased rainfall during the growing season promotes the formation of more stable helical structures, as supported by the higher relative crystallinity observed in regions with greater precipitation (Figure 4, Table 2). Across all regions, the waxy sorghum variety LN3 consistently showed higher double helix content than the non-waxy variety LZ82, which is attributed to its higher amylopectin content and lower amylose proportion. These observations are in strong agreement with the relative crystallinity trends observed via XRD, where both LN3 and GY-region samples showed the highest crystallinity. However, it is important to note that the proportion of double helices revealed by NMR was generally higher than the crystallinity measured by XRD [29]. This reflects fundamental differences in the principles of the two techniques: XRD detects long-range crystalline packing, while NMR captures local conformational arrangements, including both single and double helices [54,56]. Consequently, discrepancies between XRD-derived crystallinity and NMR-based structural components are expected and highlight the multiscale complexity of starch architecture.

Interestingly, the distribution of single helix and amorphous components did not show a consistent trend across varieties or regions. This complexity requires further investigation to fully elucidate the factors influencing these structural elements. Notably, the GY region exhibited the highest proportion of double helices and the lowest single helix content, yet paradoxically also showed the highest amorphous components. This result may suggest that while precipitation favors the formation of stable double helices, it also increases the proportion of unstructured chains that do not participate in ordered packing—possibly due to enhanced starch synthesis leading to more heterogeneous granule regions, or increased hydration that may disrupt some local interactions [12,57,58].

Overall, these findings demonstrate that starch structural organization is governed by a complex interplay between environmental factors and intrinsic molecular composition. While double helices strongly reflect genotype and precipitation, single helix and amorphous components appear to result from more nuanced molecular dynamics. The combined use of XRD and NMR provides a complementary, multiscale understanding of starch structure in sorghum.

### 3.7. Thermal Properties and Pasting Behavior of Sorghum Starch Under Environmental Variation

**Figure 6:**
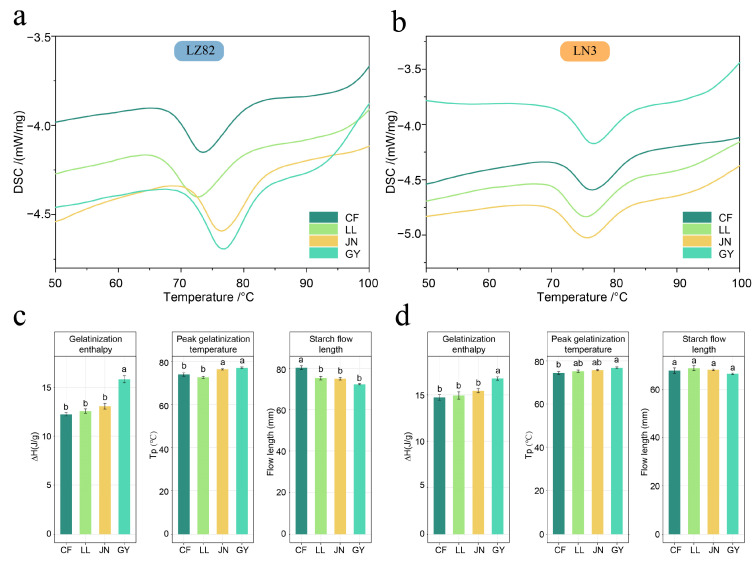
Thermal properties and flow behavior of starch samples from different regions. (**a**,**b**) Gelatinization curves of LZ82 and LN3 starch from various regions. (**c**,**d**) Comparison of gelatinization enthalpy (∆H, J/g), peak gelatinization temperature (Tp, °C), and flow length (mm) under different environmental conditions. Different lower-case letters indicate statistical significance at *p* < 0.05.

The thermal properties of sorghum starch were characterized using key gelatinization parameters, including peak temperature (Tp) and gelatinization enthalpy (ΔH), which are widely accepted as indicators of crystalline structural stability [59,60]. In this study, both LN3 and LZ82 samples grown in the more humid regions—particularly in GY—exhibited ΔH (15.84 and 16.76 J/g) and Tp (77.07 and 76.73 °C), respectively (Figure 6, Table S8). Given that both cultivars showed significantly higher ΔH and Tp values in GY—the site with the highest relative humidity—compared with CF, the driest site (Table 2), these results suggest that sorghum starch formed under high relative humidity conditions develops more stable and well-ordered crystalline structures. This observation is consistent with previous reports highlighting the correlation between increased ΔH and enhanced crystallinity [61,62]. Moreover, within each ecological region, LN3 consistently exhibited higher ΔH than LZ82, aligning with our earlier findings of higher relative crystallinity, greater double-helix content, and increased branching degree in LN3. These structural features contribute to a more compact and thermally stable starch network. This trend is corroborated by the structural characteristics revealed through XRD and NMR analyses.

To further evaluate the functional implications of starch structure during gelatinization, paste viscosity was assessed using flow length measurements (Figure 6, Table S8). LN3 displayed consistently shorter flow lengths across all regions compared to LZ82, indicating a higher paste consistency and lower fluidity. Notably, LZ82 exhibited substantial regional variation, with the CF region showing the longest flow length, corresponding to the weakest paste viscosity. In contrast, LN3 samples showed minimal regional variation and uniformly short flow lengths. These differences may be attributed to variations in amylopectin content and chain architecture, where a denser network of short branches and stable double helices likely restricts molecular mobility, contributing to a thicker and more cohesive paste [63].

In summary, both the thermal properties and paste consistency of sorghum starch are modulated by genotype and ecological factors. The high ΔH and Tp values observed in Guiyang samples coincided with higher precipitation levels, suggesting a potential association between precipitation-related environmental conditions and starch crystalline stability. Meanwhile, the waxy variety LN3 consistently demonstrates superior thermal resistance and paste viscosity due to its more ordered molecular structure. These findings provide structural insights relevant to starch functional properties and processing applications.

### 3.8. Integrated Effects of Environmental Factors on Starch Structure and Processing Functionality in Sorghum

**Figure 7:**
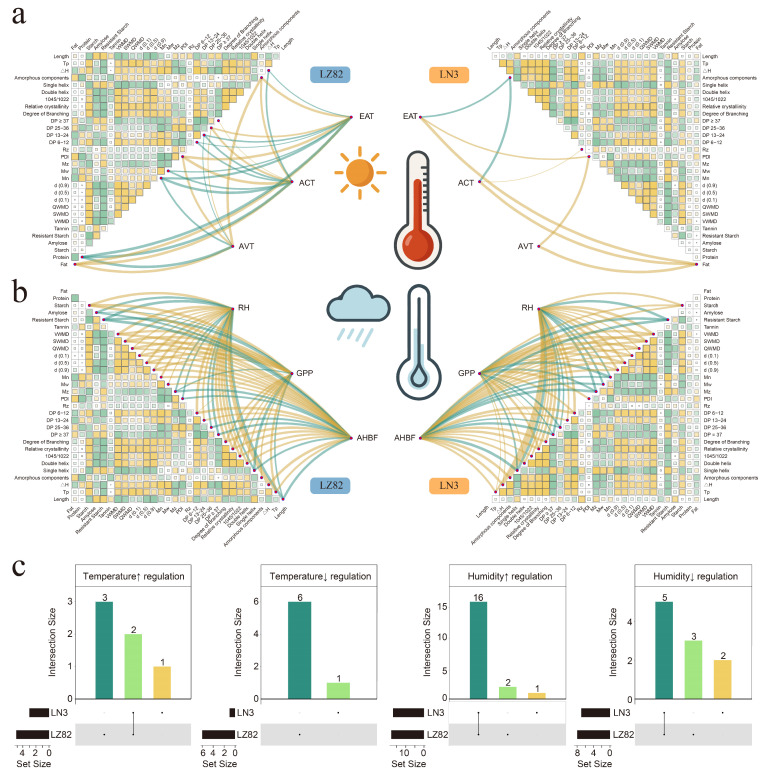
Integrated effects of environmental factors on starch structure and processing functionality in sorghum. (**a**) Correlations between temperature-related environmental factors and grain quality, starch structure, and processing traits. (**b**) Correlations between precipitation-related environmental factors and the same set of traits. The heatmap displays Pearson correlation coefficients (r) among main variables, with color gradients from green to yellow indicating the shift from negative to positive correlations. Significant correlations (*p* < 0.05) between main variables and environmental factors are represented by connecting lines, where line color indicates positive (yellow) or negative (green) regulation, and line thickness reflects the absolute correlation strength (|r|). (**c**) The upsetR plot summarizes the overlapping effects of different environmental factors on trait regulation in LZ82 and LN3. Upward and downward arrows indicate upregulation and downregulation, respectively.

**Figure 8:**
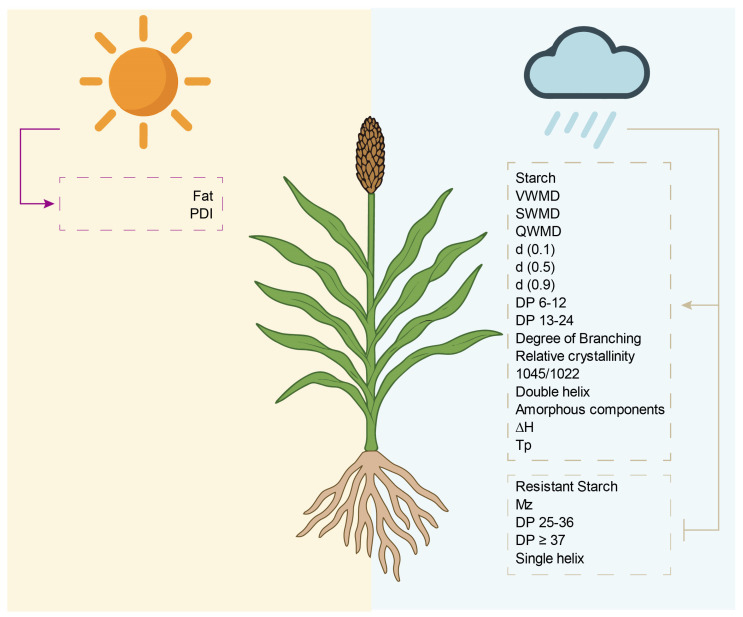
A schematic illustration outlines the integrated regulatory effects of temperature and humidity on sorghum grain quality, starch structure, and processing performance.

To further elucidate how environmental factors influence grain quality, starch structure, and processing properties, we conducted correlation analyses using the three most influential environmental variables from PC1 (temperature-driven) and PC2 (precipitation-driven) based on principal component analysis (Figure 7). As shown in Figure 7a, temperature-related factors (EGDD, GDD, and MGST) were positively correlated with PDI and fat content in both LN3 and LZ82, suggesting that elevated temperatures promote lipid accumulation and broaden molecular weight distributions (Figure 7c and Figure 8). This may be attributed to the upregulation of key lipid biosynthetic genes under moderately high temperature conditions [64], while the broader molecular weight distribution could result from temperature-induced alterations in the activities of starch synthases, branching enzymes, and debranching enzymes [65]. Interestingly, amorphous components were positively regulated by temperature in LZ82 but negatively regulated in LN3, highlighting a genotype-dependent structural response and indicating that starch molecular organization may be governed by complex genotype–environment interactions [66,67].

In contrast, precipitation factors (MGSRH, TGSP, and MPFRH) showed substantially stronger correlations with most starch-related traits than temperature (Figure 7b), showing that rainfall-related variables tended to be associated with variations in starch-related traits, indicating a possible influence of precipitation on starch architecture [68,69]. Across both genotypes, 16 starch and processing-related traits—including starch content, granule size, Mw, A/B1 chain ratio, branching degree, crystallinity, molecular order, double helices, and gelatinization temperature—exhibited significant positive correlations with precipitation (Figure 7c and Figure 8). Meanwhile, resistant starch content, B2/B3 chain ratio, and single helix content were negatively correlated, suggesting that increased precipitation favors the synthesis of more branched and crystalline starch structures while limiting the formation of long-chain linear regions (Figure 7c and Figure 8).

It is well accepted that structure determines function [70]. We further examined the associations between thermal properties (ΔH and Tp) and starch structural traits. In LZ82, ΔH and Tp were positively correlated with granule size, Mw, Mn, A/B1 chains, branching degree, crystallinity, molecular order, and double helices, but negatively correlated with resistant starch, Mz, PDI, and B2/B3 chains. In contrast, LN3 showed positive correlations between ΔH and Tp with granule size, B2/B3 chains, branching degree, crystallinity, molecular order, double helices, and amorphous regions, and negative correlations with resistant starch, Mn, Mw, Mz, and single helices. These contrasting trends suggest distinct thermodynamic adaptation mechanisms between the two genotypes: LN3 appears to rely on higher branching and conformational flexibility to maintain thermal stability, whereas LZ82 is more dependent on regular chain alignment and crystalline density. This interpretation is consistent with previous reports that starches with higher crystallinity and longer double helices generally require more energy for gelatinization temperature due to stronger molecular order and hydrogen bonding [71]. Conversely, increased branching and a more flexible amorphous matrix can enhance the mobility of molecular chains, allowing thermal adaptation without substantial disruption of crystalline regions [72].

Together, these results construct an integrated framework linking environmental conditions (temperature and precipitation) to molecular structure (chain length, branching, crystallinity) and functionality (processing and thermal behavior), offering valuable insights into the genotype-by-environment interplay shaping sorghum starch quality.

## 4. Conclusions

This study systematically elucidated the interactive effects of temperature and precipitation on sorghum starch structures and processing functionalities through multi-site field trials. Precipitation emerged as the dominant factor, facilitating amylopectin biosynthesis, branching complexity, crystalline organization, and molecular ordering, thereby enhancing thermal stability and pasting quality. Genotypic divergence between waxy LN3 and non-waxy LZ82 further shaped starch structural adaptation and environmental responses. These integrated findings offer theoretical support for precision sorghum breeding, functional starch modification, and improved utilization of sorghum resources under changing climatic conditions.

## Figures and Tables

**Table 1:** Grain nutritional composition of LZ82 and LN3 across different ecological zones.

Cultivars	Treatment	Fat (%)	Protein (g/kg)	Starch (%)	Amylose (%)	Resistant Starch (%)	Tannin (mg/g)
LZ82	CF	3.84 ± 0.10 b	8.26 ± 0.10 b	57.08 ± 0.13 c	16.24 ± 0.12 a	9.17 ± 0.14 a	13.67 ± 1.05 a
	LL	3.90 ± 0.22 b	7.88 ± 0.05 c	57.60 ± 0.57 c	15.30 ± 0.14 bc	7.30 ± 0.17 b	10.65 ± 0.82 c
	JN	4.29 ± 0.17 a	7.59 ± 0.09 d	62.24 ± 0.39 b	15.43 ± 0.10 b	3.55 ± 0.08 c	10.95 ± 0.07 bc
	GY	3.46 ± 0.05 c	8.54 ± 0.11 a	63.22 ± 0.22 a	15.15 ± 0.08 c	1.76 ± 0.05 d	12.19 ± 0.15 b
LN3	CF	3.30 ± 0.38 b	7.55 ± 0.16 a	57.30 ± 0.60 b	5.50 ± 0.76 b	2.32 ± 0.16 a	14.81 ± 2.10 a
	LL	3.45 ± 0.25 ab	8.29 ± 0.39 a	59.95 ± 1.83 ab	7.23 ± 0.62 a	1.94 ± 0.15 b	10.95 ± 0.83 b
	JN	4.01 ± 0.45 a	8.09 ± 1.23 a	61.42 ± 1.25 a	5.31 ± 0.35 b	0.92 ± 0.07 c	11.71 ± 0.42 b
	GY	2.84 ± 0.10 b	8.43 ± 1.05 a	61.84 ± 1.81 a	5.07 ± 0.21 b	0.73 ± 0.05 c	13.38 ± 1.63 ab

ANOVA was performed separately for each sorghum cultivar (LZ82 and LN3) across different locations using the general linear model (GLM). Abbreviations for locations: CF, Chifeng; LL, Lvliang; JN, Jinan; GY, Guiyang. Values in the same column with different letters are significantly different (*p* < 0.05).

**Table 2:** Environmental factors during the sorghum growing season at different locations.

Treatment	MGST (°C)	MGSRH (%)	TGSP (mm)	GSSD (h)	GDD (°C)	EGDD (°C)	MPFT (°C)	MPFRH (%)	PFP (mm)	PFSD (h)	PF-GDD (°C)	PF-EGDD (°C)
LL	23.73	60.44	235.9	969.8	2895.4	1675.4	23.59	53.88	105	308.3	1079.1	629.1
JN	26.36	65.24	585.4	849.2	3110.1	1930.1	29.11	62.42	226.7	356.5	1305.3	745.3
CF	22.79	51.74	184	951.4	2780.5	1560.5	23.18	47.68	77.4	274.3	717.4	387.4
GY	21.72	77.29	497.4	600.8	2497.5	1348.9	20.81	76.5	179.1	194.9	812.2	472.2

All meteorological data were obtained from local meteorological stations and correspond to the experimental year 2023. Climatic variables are defined as follows—mean growing-season air temperature (MGST); mean growing-season relative humidity (MGSRH); total growing-season precipitation (TGSP); growing-season sunshine duration (GSSD); growing degree days (GDD); effective growing degree days (EGDD); mean pre-flowering air temperature (MPFT); mean pre-flowering relative humidity (MPFRH); total post-flowering precipitation (PFP); post-flowering sunshine duration (PFSD); post-flowering cumulative growing degree days (PF-GDD); and post-flowering effective growing degree days (PF-EGDD).

**Table 3:** Effects of different growing environments on the molecular weight distribution parameters of two sorghum varieties.

Cultivars	Treatment	Mn (×10^7^ g/mol)	Mw (×10^7^ g/mol)	Mz (×10^7^ g/mol)	DPI	Rz (nm)
LZ82	CF	3.94 ± 0.15 e	11.77 ± 0.45 d	21.51 ± 0.40 d	2.99 ± 0.01 b	304.15 ± 23.02 ab
	LL	3.24 ± 0.10 f	11.05 ± 0.16 d	24.07 ± 0.45 b	3.41 ± 0.06 a	313.13 ± 13.53 ab
	JN	3.31 ± 0.22 f	11.56 ± 0.49 d	20.63 ± 0.49 d	3.50 ± 0.09 a	301.08 ± 16.01 ab
	GY	7.95 ± 0.19 b	12.88 ± 0.35 c	17.66 ± 0.33 f	1.62 ± 0.01 e	283.47 ± 13.68 b
LN3	CF	8.69 ± 0.21 a	15.16 ± 0.29 a	25.24 ± 0.51 a	1.75 ± 0.01 de	316.45 ± 12.16 ab
	LL	8.18 ± 0.20 b	14.24 ± 0.73 b	25.90 ± 0.35 a	1.74 ± 0.12 de	311.56 ± 19.11 ab
	JN	6.76 ± 0.23 d	14.44 ± 0.29 ab	22.45 ± 0.38 c	2.14 ± 0.04 c	323.10 ± 17.28 a
	GY	7.25 ± 0.17 c	13.30 ± 0.24 c	19.43 ± 0.41 e	1.83 ± 0.01 d	288.57 ± 13.85 ab

Data are means ± standard deviation, n = 3. Values in the same column with different letters are significantly different (*p* < 0.05).

## Data Availability

The original contributions presented in this study are included in the article/Supplementary Material. Further inquiries can be directed to the corresponding author.

## Supplementary Materials

The following supporting information can be downloaded at: https://www.mdpi.com/article/10.3390/foods14244204/s1, Figure S1. Associations between environmental principal components (PC1 and PC2) and grain nutritional traits in sorghum. (a) LZ82; (b) LN3. Figure S2. Molecular weight distribution of two sorghum cultivars grown across four ecological zones. The RI (refractive index) detector indicates the overall concentration distribution of the eluted starch molecules. The LS (light scattering) detector reflects the scattering intensity proportional to molecular weight, highlighting high-molecular-weight components. The MM (molar mass) curve represents the calculated molar mass distribution derived from the combined RI and LS signals. Table S1. Effects of different growing environments on grain size of two sorghum varieties. Table S2. Effects of different growing environments on chain-length distribution of two sorghum varieties. Table S3. Effects of different growing environments on degree of branching of two sorghum varieties. Table S4. Effects of different growing environments on amylopectin content of two sorghum varieties. Table S5. Effects of different growing environments on relative crystallinity of two sorghum varieties. Table S6. Effects of different growing environments on IR ratio of two sorghum varieties. Table S7. Relative proportions of double helix, single helix and amorphous components of starch. Table S8. Effects of different growing environments on thermal properties and pasting behavior of two sorghum varieties.
