# Supplementary material for: Influence of Environmental Factors on the Starch Quality of Sorghum: A Multifaceted Analysis of Structural, Nutritional, and Functional Profiles
Source: Foods. 2025 Dec 7;14(24):4204. doi: 10.3390/foods14244204 (PMC12732056; doi:10.3390/foods14244204)
Supplement: Supplementary file 1 [file foods-14-04204-s001.zip › foods-3950045-supplementary.pdf]

Supplementary data

Influence of Environmental Factors on the Starch Quality of Sorghum: A Multifaceted Analysis of Structural, Nutritional, and Functional Profiles

Fulai Ke †, Baizhi Chen †, Kuangye Zhang, Jiaxu Wang, Linlin Yang, Zeyang Zhao, Fei Zhang, Han Wu, Zhipeng Zhang, Feng Lu, Yanqiu Wang, Youhou Duan, Zhiqiang Liu, Jianqiu Zou and Kai Zhu *

Sorghum Research Institute, Liaoning Academy of Agricultural Sciences, Shenyang 110161, China;
fulaike1981@163.com (F.K.); chenbaizhi0307@163.com (B.C.);
zky1319577703@163.com (K.Z.);
w15640044750@163.com (J.W.); yanglinlin0330@163.com (L.Y.);
zhaozeyangzz@163.com (Z.Z.);
zhangfei19821121@163.com (F.Z.); wuhan8453@sina.com (H.W.);
zzp906@163.com (Z.Z.);
lufeng720202023@163.com (F.L.); wangyanqiu73@126.com (Y.W.);
duanyouhou@163.com (Y.D.);
baizhichen1996@gmail.com (Z.L.); jianqiuzou@126.com (J.Z.)

* Correspondence: zhukai72@163.com

† These authors contributed equally to this work.

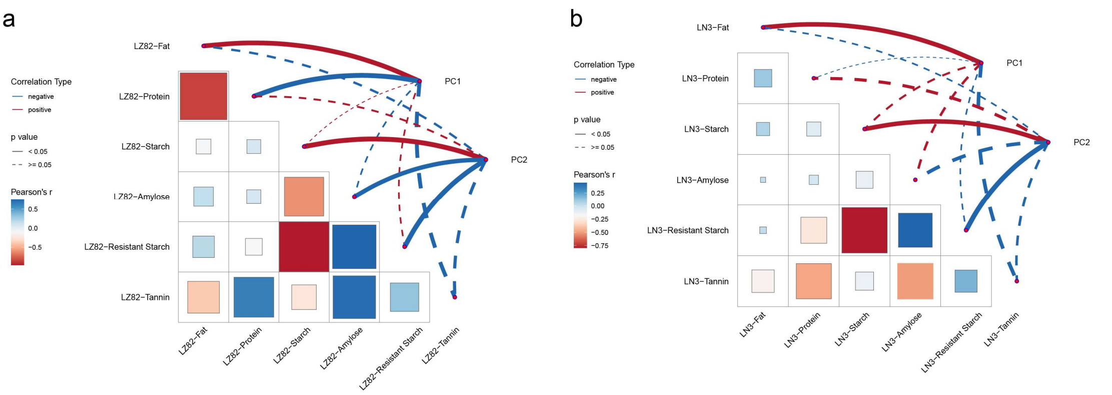

Fig.S1. Associations between environmental principal components (PC1 and PC2) and grain nutritional traits in sorghum. (a) LZ82; (b) LN3.

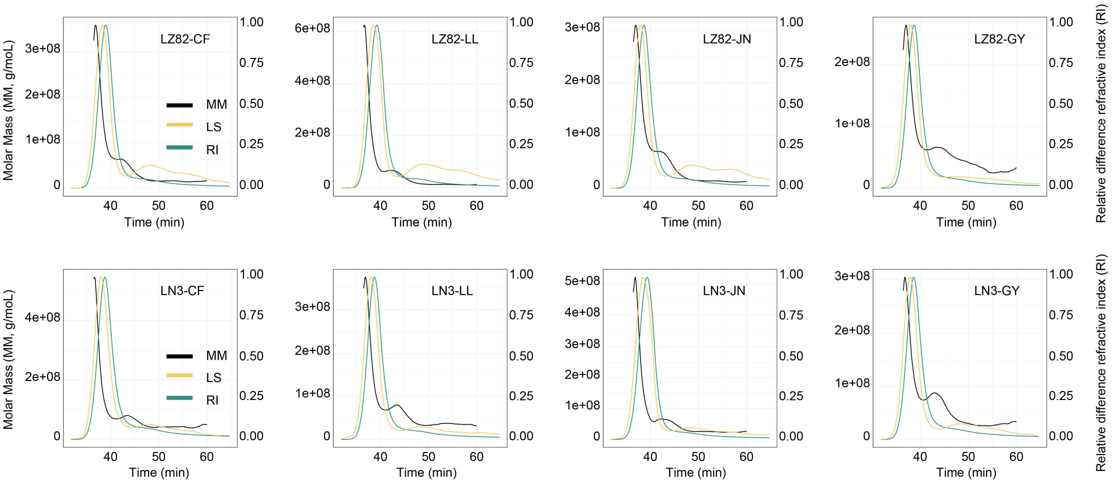

Fig.S2. Molecular weight distribution of two sorghum cultivars grown across four ecological zones. The RI (refractive index) detector indicates the overall concentration distribution of the eluted starch molecules. The LS (light scattering) detector reflects the scattering intensity proportional to molecular weight, highlighting high-molecular-weight components. The MM (molar mass) curve represents the calculated molar mass distribution derived from the combined RI and LS signals.

Table S1. Effects of different growing environments on grain size of two sorghum varieties.

Cultivars	Treatment	VWMD (μm)	SWMD (μm)	QWMD (μm)	d (0.1) (μm)	d (0.5) (μm)	d (0.9) (μm)
LZ82	CF	16.63±0.31f	15.67±0.21e	13.63±0.09d	11.43±0.17d	16.37±0.12f	22.80±0.24d
	LL	17.80±0.16e	16.10±0.16e	13.30±0.16d	11.30±0.16d	17.10±0.29e	25.47±0.37c
	JN	18.40±0.22d	17.10±0.16d	14.53±0.05c	12.07±0.21c	17.80±0.33d	25.70±0.43c
	GY	18.87±0.12c	17.03±0.17d	14.33±0.17c	12.00±0.16c	18.13±0.21d	26.83±0.57b
LN3	CF	18.90±0.16c	17.23±0.17cd	14.47±0.29c	12.07±0.12c	18.30±0.22cd	26.80±0.24b
	LL	19.77±0.12b	17.67±0.21c	14.37±0.31c	11.93±0.12c	18.73±0.25bc	29.30±0.36a
	JN	19.77±0.12b	18.23±0.29b	15.47±0.26b	13.03±0.12b	19.07±0.21b	27.40±0.24b
	GY	20.43±0.25a	18.80±0.29a	16.33±0.26a	13.67±0.17a	19.83±0.29a	28.57±0.41a

Note: The results were obtained from analysis of variance (ANOVA) conducted under the general linear model (GLM).

Table S2. Effects of different growing environments on chain-length distribution of two sorghum varieties.

Cultivars	Treatment	DP 6-12 (%)	DP 13-24 (%)	DP 25-36 (%)	DP ≥ 37 (%)
LZ82	CF	22.53±0.28g	48.33±0.21c	14.56±0.23a	14.58±0.72a
	LL	22.96±0.17fg	48.72±0.20bc	14.73±0.16a	13.59±0.53ab
	JN	23.28±0.18ef	48.35±0.13c	14.62±0.28a	13.76±0.58ab
	GY	25.42±0.33b	49.30±0.27a	13.64±0.10c	11.64±0.69cd
LN3	CF	23.64±0.10de	48.84±0.09b	14.11±0.14b	13.41±0.32ab
	LL	23.81±0.16d	48.77±0.15bc	14.01±0.10bc	13.40±0.21ab
	JN	24.65±0.23c	49.02±0.11ab	13.76±0.19bc	12.57±0.52bc
	GY	25.89±0.23a	49.35±0.31a	13.75±0.10bc	11.01±0.64d

Note: The results were obtained from analysis of variance (ANOVA) conducted under the general linear model (GLM).

Table S3. Effects of different growing environments on degree of branching of two sorghum varieties.

Cultivars	Treatment	Degree of Branching (%)
LZ82	CF	4.32±0.16f
	LL	4.72±0.16ef
	JN	4.99±0.22de
	GY	5.50±0.25cd
LN3	CF	5.64±0.28c
	LL	6.52±0.38b
	JN	6.58±0.36b
	GY	7.62±0.31a

Note: The results were obtained from analysis of variance (ANOVA) conducted under the general linear model (GLM).

Table S4. Effects of different growing environments on amylopectin content of two sorghum varieties.

Cultivars	Treatment	Amylopectin (%)
LZ82	CF	40.84±0.20d
	LL	42.31±0.35d
	JN	46.81±0.24c
	GY	48.07±0.12c
LN3	CF	51.80±1.11b
	LL	52.72±1.41b
	JN	56.12±0.85a
	GY	56.77±1.34a

Note: The results were obtained from analysis of variance (ANOVA) conducted under the general linear model (GLM).

Table S5. Effects of different growing environments on relative crystallinity of two sorghum varieties.

Cultivars	Treatment	Relative crystallinity (%)
LZ82	CF	15.52±0.15c
	LL	15.67±0.19c
	JN	16.23±0.15b
	GY	16.37±0.21b
LN3	CF	16.21±0.14b
	LL	16.45±0.19b
	JN	17.07±0.21a
	GY	17.36±0.25a

Note: The results were obtained from analysis of variance (ANOVA) conducted under the general linear model (GLM).

Table S6. Effects of different growing environments on IR ratio of two sorghum varieties.

Cultivars	Treatment	1045/1022cm^{-1}
LZ82	CF	0.88±0.03c
	LL	0.95±0.02b
	JN	0.96±0.01b
	GY	0.99±0.02ab
LN3	CF	0.94±0.02b
	LL	0.99±0.03ab
	JN	0.99±0.02ab
	GY	1.01±0.03a

Note: The results were obtained from analysis of variance (ANOVA) conducted under the general linear model (GLM).

Table S7. Relative proportions of double helix, single helix and amorphous components of starch.

Cultivars	Treatment	Double helix (%)	Single helix (%)	Amorphous components (%)
LZ82	CF	37.22±1.39e	1.62±0.73e	54.62±1.91a
	LL	42.11±1.33d	6.80±0.43b	49.89±0.84b
	JN	43.34±1.68d	12.92±0.97a	43.17±1.58cd
	GY	44.36±1.77cd	3.80±0.19d	45.33±1.16c
LN3	CF	45.35±0.78bcd	3.67±0.57d	44.98±1.52c
	LL	46.97±0.90abc	5.69±0.58bc	41.46±1.25de
	JN	48.01±1.51ab	5.37±0.79c	38.93±1.29e
	GY	49.50±1.69a	13.40±0.44a	32.34±1.15f

Note: The results were obtained from analysis of variance (ANOVA) conducted under the general linear model (GLM).

Table S8. Effects of different growing environments on thermal properties and pasting behavior of two sorghum varieties.

Cultivars	Treatment	ΔH(J/g)	Tp (℃)	Length（mm）
LZ82	CF	12.24±0.22d	74.00±1.07de	80.33±1.25a
	LL	12.58±0.32d	72.67±0.70e	75.33±1.25b
	JN	13.07±0.41d	76.37±0.41ab	75.00±0.82b
	GY	15.84±0.51b	77.07±0.60a	72.33±0.47c
LN3	CF	14.71±0.42c	74.37±0.90cd	68.00±1.63d
	LL	14.93±0.55c	75.17±0.77bcd	69.00±1.41d
	JN	15.43±0.31bc	75.67±0.42abc	68.33±0.47d
	GY	16.76±0.25a	76.73±0.61ab	66.67±0.47d

Note: The results were obtained from analysis of variance (ANOVA) conducted under the general linear model (GLM).
